# Comparative transcriptomic analysis of normal and abnormal *in vitro* flowers in *Cymbidium nanulum* Y. S. Wu et S. C. Chen identifies differentially expressed genes and candidate genes involved in flower formation

**DOI:** 10.3389/fpls.2022.1007913

**Published:** 2022-10-24

**Authors:** Shuangbin Fu, Yanping Yang, Peilong Wang, Zhen Ying, Wan Xu, Zhuang Zhou

**Affiliations:** Zhejiang Institute of Subtropical Crops, Zhejiang Academy of Agricultural Sciences, Wenzhou, China

**Keywords:** orchid, *Cymbidium nanulum* Y. S. Wu et S. C. Chen, *in vitro* flower, abnormal flower, transcriptomic analysis, WGCNA

## Abstract

It is beneficial for breeding and boosting the flower value of ornamental plants such as orchids, which can take several years of growth before blooming. Over the past few years, *in vitro* flowering of *Cymbidium nanulum* Y. S. Wu et S. C. Chen has been successfully induced; nevertheless, the production of many abnormal flowers has considerably limited the efficiency of this technique. We carried out transcriptomic analysis between normal and abnormal *in vitro* flowers, each with four organs, to investigate key genes and differentially expressed genes (DEGs) and to gain a comprehensive perspective on the formation of abnormal flowers. Thirty-six DEGs significantly enriched in plant hormone signal transduction, and photosynthesis-antenna proteins pathways were identified as key genes. Their broad upregulation and several altered transcription factors (TFs), including 11 MADS-box genes, may contribute to the deformity of *in vitro* flowers. By the use of weighted geneco−expression network analysis (WGCNA), three hub genes, including one unknown gene, mitochondrial calcium uniporter (MCU) and harpin-induced gene 1/nonrace-specific disease resistance gene 1 (NDR1/HIN1-Like) were identified that might play important roles in floral organ formation. The data presented in our study may serve as a comprehensive resource for understanding the regulatory mechanisms underlying flower and floral organ formation of *C. nanulum* Y. S. Wu et S. C. Chen *in vitro*.

## Introduction

Orchids make up one of the largest flowering families and are famous for their unique flower shape and charming colors (Duan and Yazawa, 1994b; Teixeira da Silva et al., 2007). The juvenile stage of orchids is generally long, with growth lasting several years before the flowers bloom. The ability to induce orchids to bloom *in vitro* notably reduces the time required (from years to months) to reach the mature stage of flowering (Teixeira da Silva et al., 2014). Over the last 30 years, several studies have reported a shortening of the flowering time by 2–3 years using this technique, including in orchids such as *Oncidium varicosum* (Kerbauy, 1984), *Dendrobium candidum* (Wang and Xu, 1997), and *Cymbidium niveomarginatum* (Kostenyuk et al., 1999).

**Figure 1:**
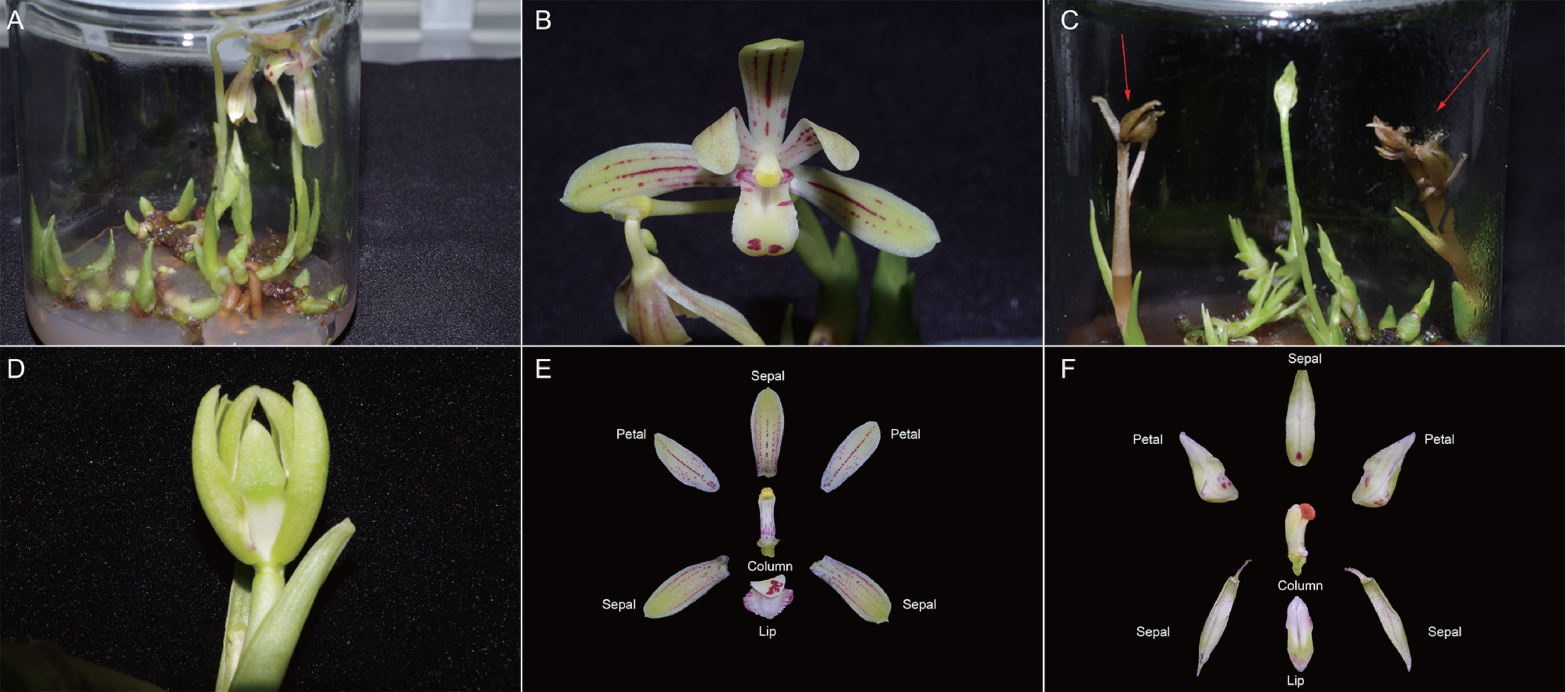
C. *nanulum* Y. S. Wu et S. C Chen flower *in vitro* and primary data reliability test. **(A, B)** Normal flowers; **(C, D)** Abnormal flowers, red arrow shows dead flowers before bloom. **(E)** The different organs of the normal flowers. **(F)** The different organs of the abnormal flowers. Generally, orchid flowers consist of three outer tepals similar to one another, two distinct inner lateral tepals, and a highly differentiated inner median tepal or lip.

According to previous research (Teixeira da Silva et al., 2014), *in vitro* flowering of *C. nanulum* Y. S. Wu et S. C. Chen (known as “Zhenzhu’ai” in China) (Wu and Chen, 1991), a short orchid with a slim distribution region and poor wild resources, was achieved by altering the culture medium (**Figures 1A**). However, most of the induced flowers were abnormal, failing to open normally and showing early apoptosis (**Figures 1C**). Although we attempted to reduce abnormal flower formation by switching to alternative media, no improvement were observed.

Data from several studies suggest that orchid flower formation is facilitated by the synchronization effects of various endogenous substances, particularly plant growth regulators (PGRs) (Kostenyuk et al., 1999; Hsiao et al., 2011). Proper PGR classes on suitable explants at appropriate doses can induce *in vitro* flower formation, especially in orchids (Teixeira da Silva, 2006). By contrast, besides the point doses or additional times may produce flowers that are unable to open regularly (Duan and Yazawa, 1994a; Teixeira da Silva et al., 2014). Transcriptomic analysis also indicated that plant hormone-related genes play key roles in the occurrence of different mutant flowers during the*ex vitro* cultivation of *Cymbidium*. For example, analysis of peloric mutant flowers and multi-tepal flowers of *C. goeringii* showed that many plant hormone-responsive genes were significantly altered in the mutant flowers (Yang et al., 2017; Shen et al., 2021). Similarly, most DEGs involved in plant hormone signal transduction implied that this process plays an important role in the development of leaf-like flower mutants in *C. ensifolium* (Wei et al., 2020).

Numerous TFs, such as MYB, bHLH, zinc finger, AP2, and SVP, have an acknowledged involvement in the control of orchid flowering (Yang et al., 2019; Ahmad et al., 2022b). Similar *in vitro* flowers, comparative transcriptome research of three orchid species revealed crucial roles that TFs played in controlling the vegetative growth, phase transition, and the quick reproductive development (Ahmad et al., 2022a). Many potential TFs were also found in the transcriptomic investigation of *C. ensifolium* and *C. goeringii*, with significant expression differences between the wild-type and the mutant (Yang et al., 2017; Wei et al., 2020).

In addition to studying the formation of the whole flower, it is necessary to explore the formation of the different flower organs. Previous studies in the model plant *Arabidopsis thaliana*, responsible for the genetic and molecular foundation of floral organogenesis, have led to the ABCDE model, which includes five primary classes of homeotic selector genes: A, B, C, D, and E (Parcy et al., 1998). Most of these key floral regulatory genes belong to the MADS-box gene family, which encode MIKC*^c^*-type MADS domain proteins that function as TFs (Theissen et al., 2000; Alvarez-Buylla et al., 2000). However, orchids are different from *A. thaliana* as they have a unique flower pattern, with a distinctive zygomorphic structure consisting of four separate organs in various shapes, including three petal-like sepals and two lateral petals, one lip, and one column organ. Aceto and Gaudio (2011) developed a special developmental-genetic code known as the”orchid code.” This hypothesis proposed that the diversity of the orchid perianth was caused by duplication events and modifications in the regulatory areas of the MADS-box genes, followed by sub- and neo-functionalization (Aceto and Gaudio, 2011). Similarly, the orchid perianth (P)-code model indicates that the higher-order sepal/petal complex determines sepal and petal formation, whereas the lip complex is exclusively required for lip formation (Hsu et al., 2015). Recent morphological observations and transcriptome analysis have summaried foral type mutations in *C. ensifolium* and found that all of them were associated with the abnormal expression of MADS-box genes. Therefore, the function of MADS-box genes in flower organ creation cannot be neglected, and it is necessary to identify homeotic genes related to flower formation in related research.

The above studies provide a general understanding of flower and abnormal flower formation; however, most of them are related to flowers produced *ex vitro*, and these results may be not suitably reproducible to produce flowers *in vitro*. Therefore, in the present study, we attempted to identify genes that may modulate the formation of abnormal flowers of *C. nanulum* Y. S. Wu et S. C. Chen *in vitro*. High-throughput Illumina RNA-seq assays were used to analyze the transcriptomes of two types of flowers. The findings of this study will contribute to a deeper understanding of the mechanisms underlying flower and floral organ formation, particularly for flower defects of *C. nanulum* Y. S. Wu et S. C. Chen, and provide practical guidance for improving *in vitro* flower technology and molecular breeding.

## Materials and methods

### Sample collection and preparation

Flowers of *C. nanulum* Y. S. Wu et S. C. Chen were produced *in vitro* by culturing rhizomes on adjusted (Murashige and Skoog) MS (Murashige and Skoog, 1962) medium with 1 mg/L 6-benzylaminopurine, 40 g/L sucrose, and 7.5 g/L agar. Different organs of the normal flowers at full bloom stage, including NC, NL, NP, NS, AC, AL, AP, and AS were sampled independently after approximately 6 months of culture in the tissue culture room (16/8 h light/dark conditions, 25°C). Similarly, different organs of abnormal flowers, including AC, AL, AP, and AS were sampled separately (**Figures 1E**).

### Library preparation for transcriptome sequencing

RNA (1 µg per sample) was used as input material for the sample preparations. Sequencing libraries were generated using the NEBNext^®^ Ultra^TM^ RNA Library Prep Kit for Illumina^®^ (NEB, USA) following the manufacturer’s instructions, and index codes were added to attribute sequences to each sample.

### Data analysis

The sequences were further processed with a bioinformatic pipeline tool, BMKCloud (www.biocloud.net) online platform.

### Transcriptome assembly

The left files (read1 files) from all libraries/samples were pooled into one big left.fq file, and right files (read2 files) into one big right.fq file. Transcriptome assembly was accomplished based on the left.fq and right.fq using Trinity (Gray and Estelle, 2000) with min_kmer_cov set to 2 by default and all other parameters set to default.

### Quantification of gene expression levels

Gene expression levels were estimated by RSEM (Li and Dewey, 2011) for each sample: 1. Clean data were mapped back onto the assembled transcriptome. 2. Read count for each gene was obtained from the mapping results.

### Differential expression analysis

Differential expression analysis of two conditions/groups was performed using the DESeq R package (1.10.1) (Anders and Huber, 2010). DESeq provides statistical routines for determining differential expression in digital gene expression data using a model based on the negative binomial distribution. The resulting P values were adjusted using Benjamini and Hochberg’s approach for controlling the false discovery rate. In the current project, the threshold for differentially expressed genes was set as FC (Fold Change) no smaller than 2 with FDR smaller than FDR <0.01. FC stands for the fold change in expression between two samples (groups).

### Unigene functional annotation

The sequences of unigenes were annotated by DIAMOND (Buchfink et al., 2015) against databases including NR, Swiss-Prot, GO, COG, KOG, eggNOG, KEGG. KEGG Orthology of unigenes were obtained by KOBAS (Bu et al., 2021). The amino acid sequences of unigenes were predicted and the predicted sequences were annotated by blasting against Pfam (Finn et al., 2008) database by HMMER (Eddy, 1998). In this study, with threshold of BLAST E-value no larger than 1e-5 and HMMER E-value on larger than 1e-10.

### Gene network construction and screening of hub genes

The WGCNA R package (Langfelder and Horvath, 2008) was used to construct the co-expression networks. Genes with RPKM values <1 were removed from the samples, and the remaining genes were used for WGCNA. The hub genes were screened based on the module KME (eigengene connectivity) values and high-weight values. The correlation networks were drawn using Cytoscape 3.8.2 (Shannon et al., 2003).

### Verification by RT−qPCR

This analysis was done on RNA extracted from four flower organs. Using the TUREscript 1st Stand cDNA SYNTHESIS Kit (Aidlab), we carried out cDNA synthesis as outlined in the protocol provided by the manufacturer. RT-qPCR was performed in a 10 µL reaction mixture consisting of 5 µL of 2×SYBR^®^ Green Supermix, 1 µL of diluted template cDNA, 0.5 µL of each primer, and 3 µL of ddH_2_O. RT-qPCR was accomplished using qTOWER 2.0/2.2 Quantitative Real-Time PCR Thermal Cyclers (Germany), Analytik Jena (Germany) with the following thermocycling programme: denaturation at 95°C for 3 min, followed by 40 cycles of 95°C for 10 s, 58°C for 30 s and 72°C for 6 s. The sequences of the primers are shown in **Table S2**. We employed the *RPS3* (Luo et al., 2014) gene as the internal standardization gene. Fold gene expression differences were computed using the 2^−ΔΔCT^ approach (Livak and Schmittgen, 2001).

## Results

### Clean data statistics

Twenty-four samples, including NC, NL, NP, NS, AC, AL, AP, and AS, have been processed for mRNA sequencing. A total of 158.07 Gb clean data were obtained with a minimum size of 5.73 Gb for each sample, and the percentage of bases with a quality score of 30 was higher than 94.54%. The clean data of all samples are summarized in **Supplementary Table S1**.

### *De novo* transcriptome assembly

**Figure 2:**
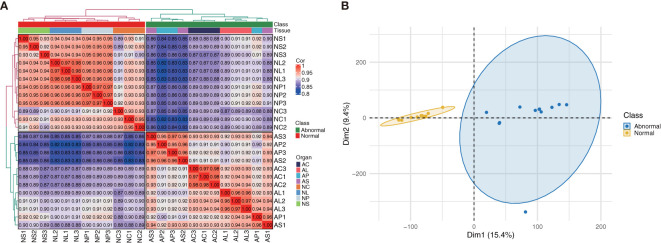
**(A)** Heatmap showing correlations between samples (Spearman method). **(B)** PCA analysis of *C.* *nanulum* Y. S. Wu & S. C. Chen flower produced *in vitro*.

Sequence assembly results showed 59,444 unigenes. The N50 value for unigenes was 1,865. The completeness of the assembly was very high. The distribution of assembled transcripts and unigenes is shown in **Supplementary Figure S1**. The density plots and box plots presented in RNA-Seq can provide a highly sensitive estimation of gene expression based on the FPKM values of all genes in each sample, thus verifying data reliability (**Supplementary Figure S2**). The correlation coefficients of samples within and between groups were calculated, and a heat map was created. The R2 value for the correlation coefficient of each sample was> 0.8 (**Figure 2**); normal and abnormal flowers were classified clearly, although AP and AS were not significantly classified. This was confirmed by subsequent PCA (Principal Component Analysis), which showed that normal and abnormal flowers were classified clearly, whereas flower organs were difficult to distinguish (**Figure 2**). Therefore, these samples could be used to identify DEGs.

### Gene expression analysis

**Figure 3:**
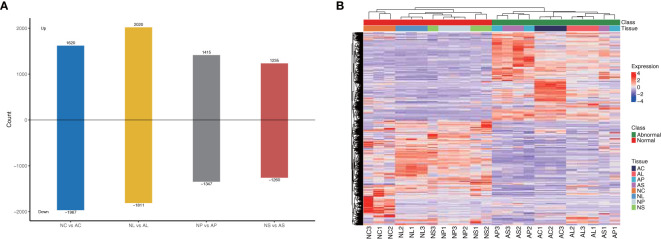
DEGs between different comparisons. **(A)** The number of up-regulated and down-regulated genes between NC *vs* AC, NL *vs* AL, NP *vs* AP, NS *vs* AS. **(B)** Heatmap of DEGs, scaled by rows.

We subjected the expression values to pairwise comparisons (i.e., NC *vs* AC, NL *vs* AL, NP *vs* AP, and NS *vs* AS) to identify the DEGs between normal and abnormal flower organs. The analysis showed 1,620 upregulated and 1,967 downregulated DEGs for the comparison between NC and AC, 2,020 upregulated and 1,811 downregulated DEGs for the comparison between NL and AL, 1,415 upregulated and 1,347 downregulated DEGs for the comparison between NP and AP, and 1,235 upregulated and 1,260 downregulated DEGs for the comparison between NS and AS (**Figure 3**). By contrast, the DEGs obtained when comparing NP and AP, and NS and AS were fewer than those obtained when comparing NC and AC, and NL and AL. The heatmap (**Figure 3**) showed that the DEGs among different organs were divided into two (normal and abnormal) groups. Significant and wide differences between normal and abnormal flowers were also observed, as shown in **Figure 2**.

### KEGG enrichment analysis of DEGs

**Figure 4:**
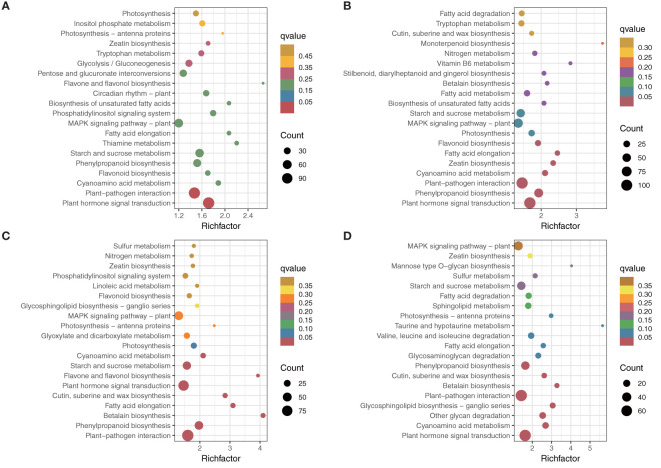
KEGG enrichment analysis of different comparisons. **(A)** NC *vs* AC. **(B)** NL *vs* AL. **(C)** NP *vs* AP. **(D)** NS *vs* AS.

Among the top 20 most enriched KEGG pathways shown in **Figure 3**, plant hormone signal transduction (KEGG 04075) and plant–pathogen interaction (KEGG 04626) were the two most significantly enriched KEGG pathways among all the comparisons (**Figures 4D**). Additionally, starch and sucrose metabolism, phenylpropanoid biosynthesis, and MAPK signaling pathways were also significantly enriched among different comparisons.

**Figure 5:**
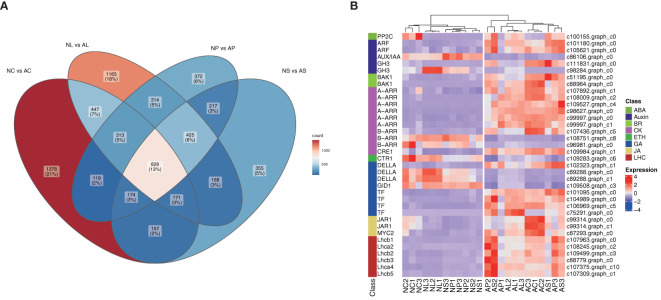
Screening of key genes. **(A)** Venn diagram for the four comparisons. **(B)** Annotation of key genes involved in plant hormone signal transduction and of all genes involved in the photosynthesis-antenna protein pathways shown in the heatmap, scaled by rows. LHC, light-harvesting chlorophyll protein complex.

Venn diagrams were used to assess the common DEGs due to the commonality of the four comparisons (**Figure 4**). The results showed 828 DEGs in 4 comparisons (**Figure 5**) that were significantly enriched in plant hormone signal transduction and photosynthesis-antenna proteins (**Figure 5**; **Supplementary Figure S3**). Subsequently, these significantly enriched DEGs were annotated by the database and used to draw a heatmap, which indicated that most of the DEGs in the plant hormone signal transduction pathway and all those in the photosynthesis-antenna proteins pathway were upregulated compared with normal flowers.

### Transcription factors

**Figure 6:**
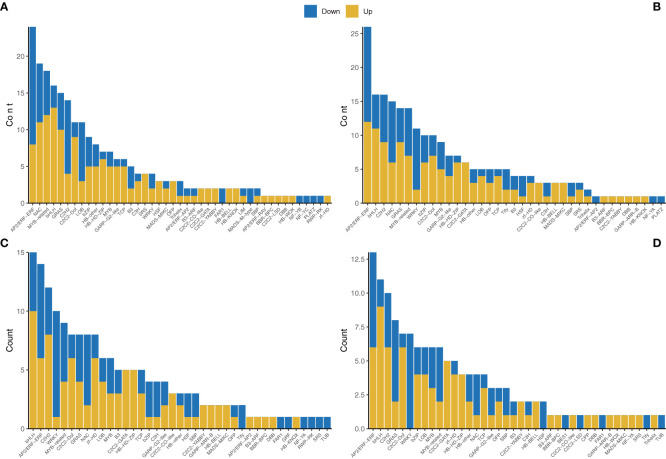
Transcription factor annotations of the unigenes of different comparisons. **(A)** NC *vs* AC. **(B)** NL *vs* AL. **(C)** NP *vs* AP. **(D)** NS *vs* AS.

A total of 771 TFs were found to have significantly changed among the different comparisons. There were 232 DEGs encoding TFs for the NC *vs* AC comparison, with 141 upregulated and 91 downregulated, 227 DEGs encoding TFs for the NL *vs* AL comparison, with 132 upregulated and 95 downregulated, 171 DEGs encoding TFs for the NP *vs* AP comparison, with 95 upregulated and 76 downregulated, and 141 DEGs encoding TFs for the NS *vs* AS comparison, with 82 upregulated and 59 downregulated. The major TF families included AP2/ERF-ERF, bHLH, C2H2, MYB, and WRKY (**Figure 6**) for all the comparisons. Furthermore, 11 unigenes (8 categories after eliminating duplication) were identified as MADS-box genes and were mostly upregulated in abnormal flower organs. We constructed a phylogenetic tree (Yu et al., 2017) to categorize the unigenes and clarify the classes of these MADS-box genes, as shown in **Supplementary Figure S4**. c107150.graph_c4 was clustered with floral homeotic genes from the B-class, c89455.graph_c2 was clustered with floral homeotic genes from the C-class, and c109210.graph_c1 was clustered with floral homeotic genes from the E-class, according to Chen and Chin’s summary (Chen and Chin, 2021) (**Supplementary Figure S4**).

### Weighted gene co−expression network analysis

**Figure 7:**
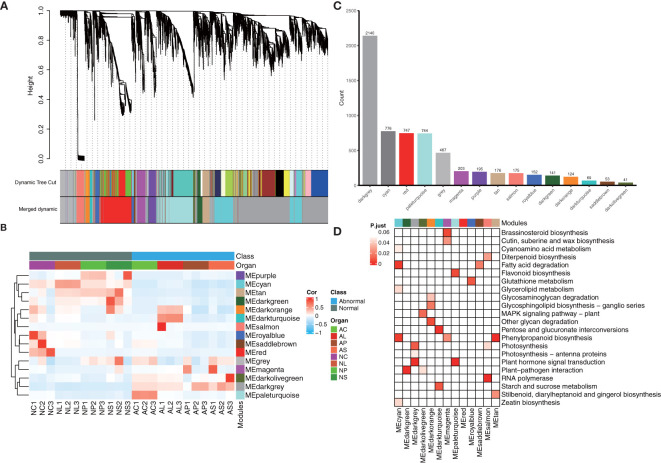
WGCNA analysis of 24 samples. **(A)** Hierarchical clustering tree showing the co-expression modules identified by WGCNA. **(B)** Heatmap of samples’ expression patterns. The color of each cell at the row–column intersection indicates the expression level of the module. **(C)** Number of genes in different modules. **(D)** KEGG enrichment analysis of the WGCNA modules. All the KEGG terms with a q-value < 0.05 are displayed.

WGCNA was used to reveal the connected gene sets that are associated with different flower organs. The network contained 6,203 genes and was divided into 15 modules (**Figures 7A**). The dark grey module had the largest number of genes (2,140), whereas the dark olive-green module had the smallest number of genes (41) (**Figure 7**). Three identified modules were found to be specifically related to normal and abnormal flower tissues; the cyan module with 776 genes, pale turquoise module with744 genes, and dark turquoise module with 69 genes were significantly positively correlated with NL1, 2, 3, AC1, 2, 3, and AL1, 2, 3, respectively (**Figure 7**; **Supplementary Figure S5**). KEGG classification showed these genes are more enriched in metabolism, besides plant hormone signal transduction and plant–pathogen interaction (**Supplementary Figure S6**). Furthermore, KEGG enrichment analysis showed that genes in the cyan module were significantly enriched in the cyanoamino acid metabolism, fatty acid degradation, glycerolipid metabolism, phenylpropanoid biosynthesis, and zeatin biosynthesis pathways. Genes in the dark turquoise module showed that they were significantly enriched in the starch and sucrose metabolism and pentose and glucoronate interconversions pathways. Genes in the pale turquoise module showed that they were significantly enriched in plant hormone signal transduction and flavonoid biosynthesis (**Figure 7**).

### Identification of hub genes and network construction

**Figure 8:**
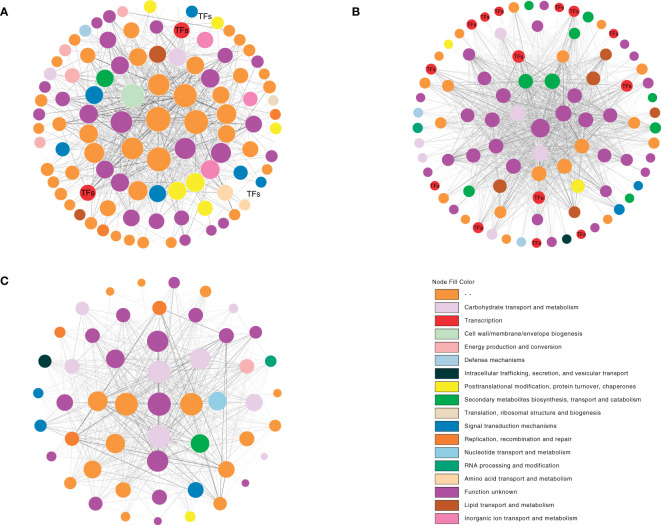
Co-expression network analysis of the cyan, pale turquoise, and dark turquoise modules. The depth of the node fill size represents the degree. The depth of the edge color represents the weight. **(A)** cyan module. **(B)** pale turquoise module. **(C)** dark turquoise module.

The top 1,000 pairs between two genes in each module (pairs >1,000) were chosen to design a gene correlation network to find hub genes from the major modules based on weight values. A hub gene was defined as the gene with the most connections. After the selection process, there were 108 genes in the cyan module, 91 genes in the pale turquoise module, and 52 genes in the dark turquoise module. According to the eggNOG database, functional categories were annotated. The results showed several unannotated and “function unknown” unigenes among the three modules, while behind them were certain “metabolism and signal transduction”-related genes such as “carbohydrate transport and metabolism”-related genes and “signal transduction mechanisms”-related genes. Notably, there were several TFs among the cyan and pale turquoise modules; nr database annotation showed these TFs to be mainly plant hormone and MADS-box-related, respectively (**Supplementary File 1**). Furthermore, according to the nr database annotation, unigene c108053.graph_c3 (unknown gene) was in the core position in the cyan module (i.e., the degree calculated by cytoscape was at the top). Unigene 109304.graph_c15 (NDR1/HIN1-Like) was in core position in the pale turquoise module. Inthe dark turquoise module, unigene c101834.graph_c0 (Calcium uniporter) was in the core position (**Figures 8C**).

### Validating the gene expression patterns by RT-qPCR

**Figure 9:**
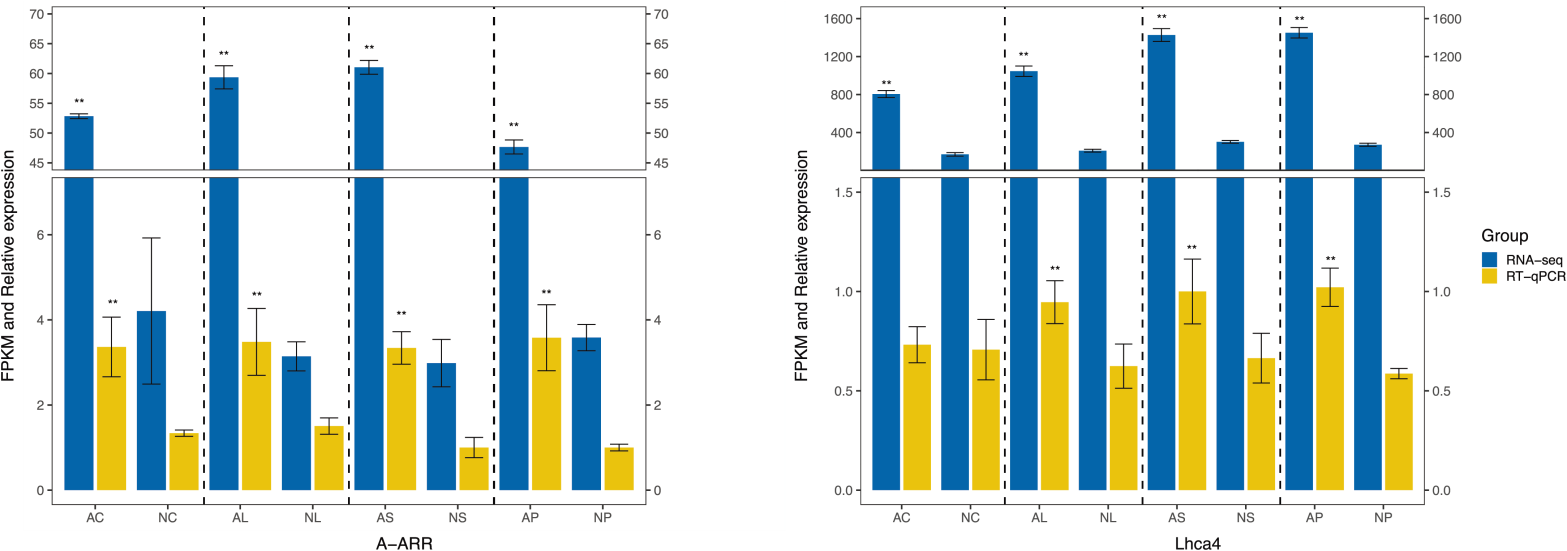
RT-qPCR-based verification of the RNA-seq analysis results. Asterisk represents statistical significance.

To further validate the reliability of the RNA-seq results, RT-qPCR was conducted to examine the expression levels of some DEGs, including 2 genes (**Supplementary Table S2**). As shown in **Figure 9**, the results of RT-qPCR were well in accordance with the expression data obtained by RNA-Seq. The expression of all these 2 genes in *C. nanulum* Y. S. Wu et S. C. Chen exhibited similar patterns among different organs.

## Discussion

### Plant hormones

KEGG annotation of the four comparisons and of common genes revealed similar gene expression patterns that were mainly mapped onto the plant hormone signal transduction pathway. These results indicated that plant hormones play key roles in the formation of normal and abnormal flowers. Several studies have similarly emphasized the role of plant hormones in flower bud differentiation and floral organ development (Kurokura et al., 2013). Among the various hormones, auxin has a regulatory function by influencing the fundamental processes of cell division, growth, and differentiation, at least to some extent. In the model plant *Arabidopsis*, *ARF6* and *ARF8* are expressed in different flower tissues and facilitatethe transition from closed bud to mature fertile bloom (Nagpal et al., 2005; Wu et al., 2006). In *C. goeringii*, peloric mutant flowers and multi-tepal flowers showed downregulation of *ARF* genes (Yang et al., 2017; Shen et al., 2021). However, in our study, *ARF* genes were all upregulated in abnormal flowers, suggesting that the mechanism underlying the formation of abnormal flowers *in vitro* differs from that of mutant flowers in nature. The expression of *AUX/IAA* and *GH3* was suppressed by AUX/IAA-ARF heterodimers generated at low auxin concentrations, while at high auxin concentrations, AUX/IAA repressors dissociate from ARFsand degrade rapidly (Gray and Estelle, 2000; Worley et al., 2000). Combining the two characteristics, normal flowers may be in a state of high auxin concentrations with the ARF activity inhibited, whereas the opposite occurs in abnormal flowers.

Benzylaminopurine, a synthetic cytokinin, promotes blooming in Orchidaceae *in vitro* and *in vivo* (Sakai and Ichihara, 2010; Teixeira da Silva et al., 2014). Evidence from transcriptome analysis also suggests that the cytokinin-responsive genes are dynamic in flower formation and development in a variety of orchids (Sun et al., 2016; Yang et al., 2017; Shen et al., 2021). Among the selected key genes in our study, all *A-ARR* genes were upregulated, whereas most *B-ARR* genes were downregulated. These findings are similar to previous studies, wherein B-ARRs were shown as positive and A-ARRs as negative regulators in a feedback loop within the cytokinin signaling circuitry (Hwang and Sheen, 2001; Kakimoto, 2003; To and Kieber, 2008). These unexpected modifications in cytokinin signaling may cause abnormal flower phenotypes.

Compared to other hormones, gibberellin is mainly associated with flower bud differentiation (Lang, 1957; Liu et al., 2017). Prior studies have noted the importance of GA3 biosynthesis, signal transduction, as well as external spray in flower formation, which significantly elevate the quality and quantity of inflorescences in orchids and many other plants (Matsumoto, 2006). In the GA3 signal transduction pathway, the GID1 protein is a soluble GA receptor, whereas DELLA domain proteins are transcriptional regulators that respond to GA and are GA-induced repressors of growth and flowering (Wu et al., 2006). In the present study, 3 *DELLA* genes were downregulated and 1 was upregulated in abnormal flowers; these results appeared in contrast to earlier findings. *DELLA* genes also showed mixed expression in peloric mutant flowers of *C. goeringii*, indicating that this phenomenon could be specific in mutant orchid flowers (Shen et al., 2021). Overall, these altered genes revealed a critical role of gibberellin in the regulation of floral patterning.

Furthermore, hormones do not control development *via* linear pathways but *via* complex interconnected webs of cross-regulation and crosstalk (Kuppusamy et al., 2008; Chandler, 2009). JA biosynthesis is promoted by GA biosynthesis during stamen, filament, and anther growth in *Arabidopsis*. GA activates—*via* the repression of DELLA—the JA biosynthesis gene *DAD1*, which in turn regulates the transcription factors MYB21, MYB24, and MYB57 (Cheng et al., 2009). Many gene targets of *SEP3* are involved in the signaling and homeostasis of several different hormones, including auxin, GA, and BR (Kaufmann et al., 2009), making *SEP3* a node for hormone cross-regulatory networks. The findings of the present study combined with those of previous studies suggest that flowering is a complex process, involving many hormones directly or indirectly.

### LHC

Another important part of key genes focuses on *LHC*, a set of several membrane proteins encoded by the nuclear genome. There are 10 different classes of nuclear-encoded *Lhc* genes, which encode 10 abundant LHC proteins in higher plants (Jansson et al., 1992) (ko00196). Apart from absorbing sunlight and transferring the excitation energy (as antenna proteins), several members of the Lhc family are also involved in the regulation of plant growth and development. For example, *Arabidopsis Lhcb* genes are implicated in ABA-induced seed germination and post-germination growth (Liu et al., 2004). Compared to the wild type, *Lhcb1* downregulation resulted in somewhat smaller and paler leaves with lower chlorophyll content (Pietrzykowska et al., 2014).

*Lhc* genes also play important roles in plant stress response and the regulation of plant stress tolerance. *Lhcb1* expression is upregulated in *Apium graveolens* under cold, heat, salt, and drought stress (Jiang et al., 2014). In rice seedlings, expression levels of *Lhca*1-4 are reduced under Fe deficiency, impairing the plant’s light-harvesting capacity and resulting in decreased photosynthetic efficiency (Yadavalli et al., 2012). In *Arabidopsis*, *Lhcb*1–6 respond to stomatal movement and are involved in ABA signaling by partly altering ROS homeostasis (Xu et al., 2012), which plays a key role in plant stress resistance. Therefore, our findings showing upregulation of *Lhca* and *Lhcb* genes appears to be perplexing. Either the upregulation of these genes affects the formation of abnormal flowers, or the abnormal flowers themselves are in a state of adversity, resulting in gene upregulation. Further experiments should be performed to validate these assumptions.

### TFs and MADS-box genes

In the model plant *A. thaliana*, flower formation and development are controlled by complex and intricate gene regulatory networks of transcription factors. In their review, Wils and Kaufmann (Wils and Kaufmann, 2017) mention that various TF families, including AP2/ERF, bHLH, MYB, MADS-box, and NAC, have been reported to be involved in floral development (Becker and Theißen, 2003; Ning et al., 2015; Matías-Hernández et al., 2016). Therefore, assessing the expression levels of TF genes in normal and abnormal flowers is useful to clarify the complex reasons for the formation of abnormal flowers. In the present study, several flower development-related TF families were identified, including AP2/ERF-ERF, bHLH, C2H2, MYB, and WRKY. The AP2/ERF-ERF family was markedly altered between normal and abnormal flowers, indicating that it plays an important role in the formation of abnormal flowers. Furthermore, the WGCNA analysis showed that some of these TFs participated in the core regulatory network (**Figures 8A**). These findings were consistent with prior studies on orchids, wherein the leaf-like flower mutant in *C. ensifolium* and multi-tepal flowers in *C. goeringii* also showed many changes of TFs (Yang et al., 2017; Wei et al., 2020). Collectively, we can infer that these significantly altered TFs play crucial roles in flower formation and development *in vitro*.

A review by Chen and Chin reported that MADS-box regulators showed diversities among different orchid genera, with modifications in the expression domains of the several classes (Chen and Chin, 2021). For example, in *Arabidopsis*, the determination of petal and stamen identity is controlled by MADS-box genes from the B-class (Fornara et al., 2003), whereas in the orchid *Dendrobium* crumenatum, the B-class genes *DcOAP3A* and *DcOPI* are expressed inthe tissues of all floral organs (Xu et al., 2006). In several *Cymbidium* mutant flowers, such as *C. goeringii*, B-class genes *CgDEF1* and E-class genes *CgSEP2* and *CgAGL6-1* are upregulated in the sepals and petals but dramatically downregulated in the lip (Xiang et al., 2018). Similarly, transcriptomic analysis shows MADS-box modifications in *C. nanulum* Y. S. Wu & S. C. Chen. For example, the B-class gene (c107150.graph_c4) was expressed in all flower organs and was upregulated in all abnormal organs except in the abnormal column. The E-class gene (c109210.graph_c1) was expressed in all flower organs and was specially upregulated in the abnormal lip. Notably, the C-class gene (c89455.graph_c2) was especially expressed in the column, in agreement with prior studies (Mondragón-Palomino and Theißen, 2008; Ai et al., 2021), which showed that C-class genes were only expressed in the column in the wild type. In addition, different from the above mutant flowers, the organs of abnormal flowers were intact (no organ switch, appearing multi-tepal or peloric; **Figure 1**). Therefore, the significant change of MADS-box genes might have contributed to the occurrence of abnormal flowers and flower organs *in vitro* rather than loss of genes. Moreover, this result proves that the formation of abnormal flowers *in vitro* has its own uniqueness.

### WGCNA and hub genes

The WGCNA results showed that not all organs are strongly related to identified modules. Only three of the identified modules were found to be specifically related to normal or abnormal flower tissues: NL, AC, and AL. Database annotation showed that genes in these modules were diverse and were not only restricted to signal transduction. They included many unknown genes, TFs, as well as metabolism and biosynthesis genes (**Figures 7**, **8**). On one hand, this result indicated that the formation of flowers and flower organs *in vitro* is very complicated and involves many different mechanisms. On the other hand, we may infer those abnormal flowers *in vitro* are not only deformed in flower patterns, but that other aspects, such as flower color or flower fragrance, may have also changed.

Based on the correlation networks map, three genes were identified as the candidate hub genes. However, unigene c108053.graph_c3 (unknown gene), unigene c109304.graph_c15 (NDR1/HIN1-Like), and unigene c101834.graph_c0 (MCU) were not TFs or genes related to flower formation.

According to previously published literature, NDR1/HIN1-like (*NHL*) genes in *Arabidopsis* include harpin-induced gene 1 (*HIN1*) and nonrace-specific disease resistance gene 1 (*NDR1*). *HIN1* is induced by harpin protein and plays an important role in various plant defense responses. Similarly, *NDR1* has been found to function in multiple plant disease resistance responses (Century et al., 1997). *HIN1* is also closely related to leaf- and flower-senescence. In tobacco, *HIN1* transcript levels peaked in the most senescent phases. Among the different flower organs, the transcripts were found highly abundant in the pistils and petals, at lower levels in the sepals and stamens, but hardly detectable in the ovules (Takahashi et al., 2004). Here, as candidate hub gene, c109304.graph_c15 showed upregulation in all abnormal flower organs, contacting the subsequent status of the abnormal flower which exhibited apoptosis (**Figure 1**), its function would be confirmed.

In animals, the MCU complex is involved in a variety of physiological processes, including cell death, muscle contraction, and several Ca^2+^-dependent stress and developmentally regulated activities (Marchi and Pinton, 2014; Kamer and Mootha, 2015). Three MCU isoforms have been found in the model plant *A. thaliana* (AtMCU1-AtMCU3). Under restricted circumstances, AtMCU1 mutants show lower root development and affect Ca^2+^dynamics (Teardo et al., 2017). In the *Arabidopsis* pollen tube germination and growth processes, the absence of AtMCU2 resulted in decreased pollen grain germination (Selles et al., 2018). The relevance of controlled MCU transport for growth and reproduction is highlighted by these findings. However, plant studies were limited to *A. thaliana*, and it is uncertain whether a similar function exists in other plants. It is also difficult to say why this gene is linked to abnormal lip development, and further follow-up studies are needed.

## Conclusions

In the current study, four-organed normal and abnormal flowers were compared using transcriptome analysis. A total of 36 candidate genes significantly enriched in plant hormone signal transduction, and photosynthesis-antenna proteins pathways were identified. The deformity of flowers may be influenced by their widespread upregulation and a number of altered transcription factors, including 11 MADS-box genes. Using WGCNA, three hub genes, including one unknown gene, MCU and NDR1/HIN1-Like were identified that might play important roles in floral organ formation.

## Data availability statement

The datasets presented in this study can be found in online repositories. The names of the repository/repositories and accession number(s) can be found below: https://ngdc.cncb.ac.cn/, PRJCA010891.

## Author contributions

SF and ZZ designed the experiments. SF, YY, YZ, XW and PW performed all experiments. SF analysed the data and wrote the manuscript. ZZ and PW revised the manuscript. All authors contributed to the article and approved the submitted version.

## Funding

This research was funded by the Wenzhou Agricultural New Variety Breeding Cooperative Group Project (grant no. 2019ZX004-3).

## Conflict of interest

The authors declare that the research was conducted in the absence of any commercial or financial relationships that could be construed as a potential conflict of interest.

## Publisher’s note

All claims expressed in this article are solely those of the authors and do not necessarily represent those of their affiliated organizations, or those of the publisher, the editors and the reviewers. Any product that may be evaluated in this article, or claim that may be made by its manufacturer, is not guaranteed or endorsed by the publisher.
