# Supplementary material for: Comparative transcriptomic analysis of normal and abnormal in vitro flowers in Cymbidium nanulum Y. S. Wu et S. C. Chen identifies differentially expressed genes and candidate genes involved in flower formation
Source: Front Plant Sci. 2022 Oct 24;13:1007913. doi: 10.3389/fpls.2022.1007913 (PMC9638074; doi:10.3389/fpls.2022.1007913)
Supplement: Supplementary file 1 [file DataSheet_1.zip › Supplementary.PDF]

frontiers

Supplementary Material

1 SUPPLEMENTARY TABLES AND FIGURES

For more information on Supplementary Material and for details on the different file types accepted, please see the Supplementary Material section of the Author Guidelines.

Figures, tables, and images will be published under a Creative Commons CC-BY licence and permission must be obtained for use of copyrighted material from other sources (including re-published/adapted/modified/partial figures and images from the internet). It is the responsibility of the authors to acquire the licenses, to follow any citation instructions requested by third-party rights holders, and cover any supplementary charges.

1.1 Table

Table S1. Clean data statistics

Samples ID	Read Number	Base Number	GC Content	%≥Q30
AC1	23,696,543	7,091,804,272	45.86%	94.88%
AC2	23,152,836	6,926,633,504	46.07%	94.61%
AC3	22,022,424	6,581,272,468	45.89%	95.73%
AL1	19,720,377	5,896,286,050	44.96%	95.64%
AL2	22,220,762	6,648,721,348	46.03%	95.43%
AL3	21,384,044	6,400,221,260	46.04%	95.56%
AP1	22,990,202	6,876,534,150	45.89%	95.87%
AP2	22,738,727	6,793,788,800	47.38%	96.21%
AP3	19,766,474	5,915,709,714	46.03%	96.01%
AS1	20,322,524	6,081,258,784	45.96%	95.76%
AS2	21,370,322	6,393,386,002	45.90%	95.77%
AS3	22,266,578	6,654,333,636	45.81%	95.84%
NC1	19,176,675	5,733,339,666	46.03%	95.78%
NC2	23,480,748	7,025,935,594	46.57%	95.27%
NC3	23,181,157	6,936,754,258	46.69%	94.99%
NL1	24,136,039	7,218,778,938	45.78%	95.13%
NL2	23,817,027	7,125,753,622	45.65%	94.54%
NL3	24,060,299	7,196,089,816	45.96%	94.98%
NP1	19,298,960	5,768,374,074	46.10%	95.75%
NP2	21,251,896	6,346,561,042	46.10%	95.83%
NP3	23,035,689	6,890,150,014	46.23%	95.65%
NS1	20,667,774	6,186,062,354	46.04%	95.35%
NS2	23,953,624	7,162,731,164	46.27%	94.88%
NS3	20,794,490	6,223,600,380	45.68%	95.85%

1.2 Figures

Table S2. Sequences of primers used for RT-qPCR verification

Gene name	Primer sequence
RPS3-1-F	GCTTCGTCTTGCGATCTTAA
RPS3-1-R	CCTTGCGGATGAATACATTCT
A-ARR-F	CAGTAGCAGCAGAAGCAGTAA
A-ARR-R	GAGAATCTCAGCCTTGTCTTCTT
Lhca4-F	TTCTATTAGACAACCGACACAGC
Lhca4-R	CTTGCTCCCTCTCTTAAAGAACTT

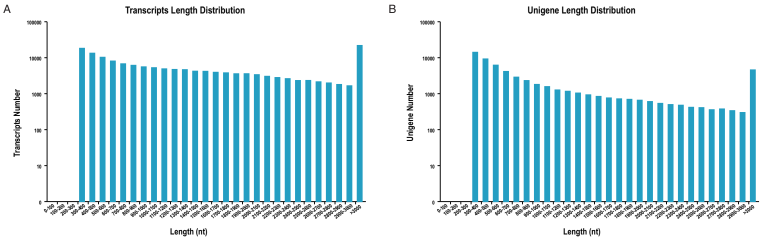

Figure S1. Length distribution of transcripts and unigenes.

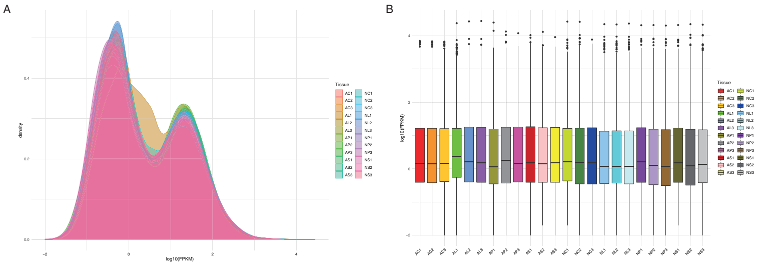

Figure S2. Distribution of gene expression level. **(A)** Distribution of FPKM density in each sample. Curves in different colours represent different samples. X-axis is log10 (FPKM) and Y-axis stands for the probability density. **(B)** FPKM box plots of each sample. X-axis stands for different samples. Y-axis is the log10 FPKM. This figure presented the overall expression level and dispersion of expression in each sample.

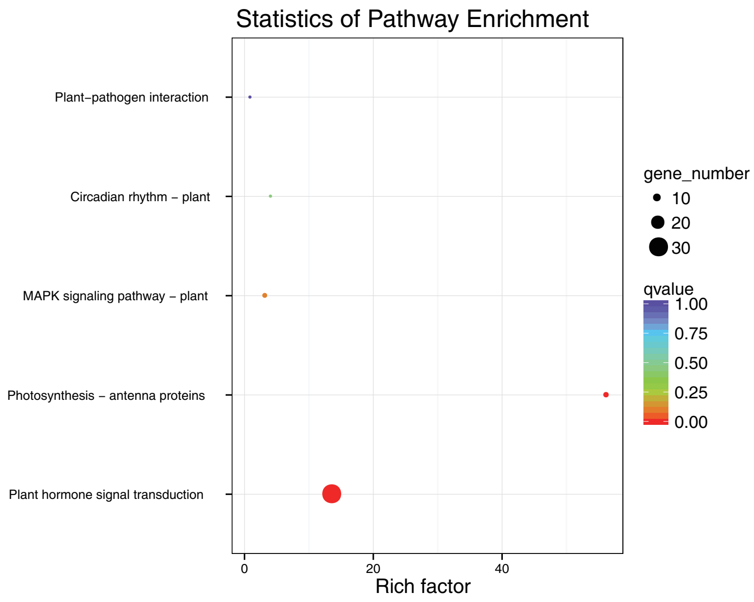

Figure S3. KEGG enrichment of key genes.

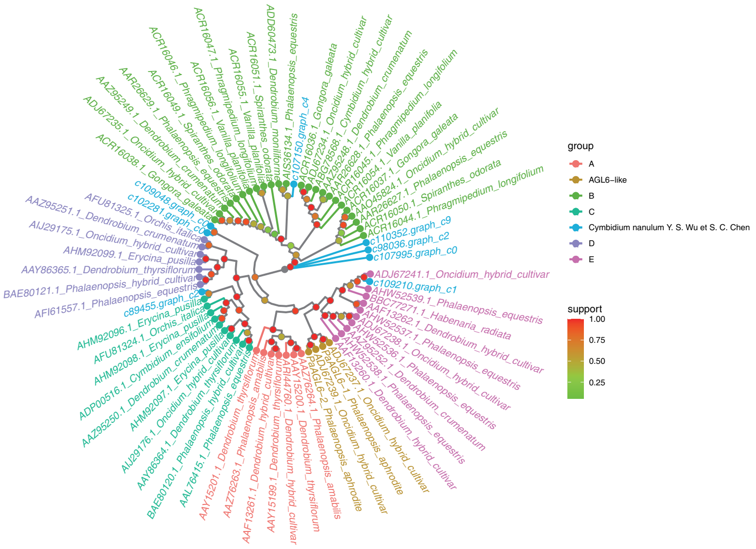

Figure S4. Phylogenetic analysis of the MADS-box transcription factors from different orchid plant species.

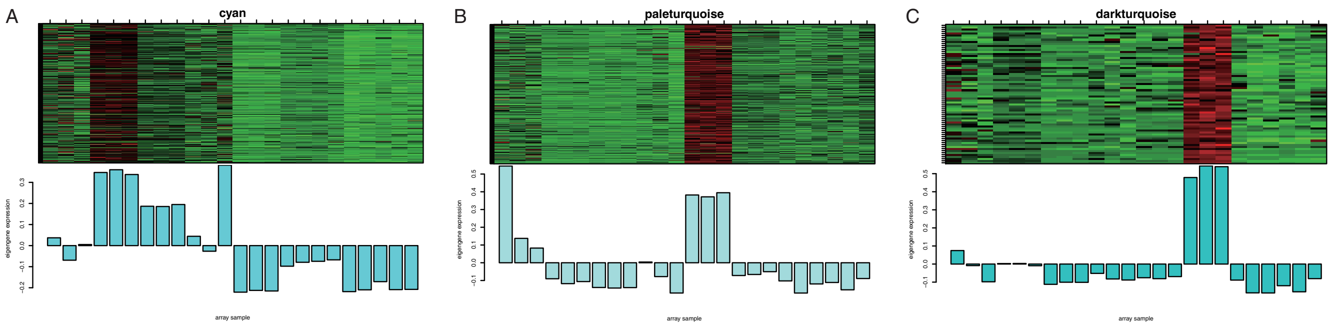

Figure S5. Heatmap showing the expression profiles of the transcripts within the **(A)** cyan module, **(B)** paleturquoise module and **(C)** darkturquoise module. The corresponding bar graphs showing the eigengene expression levels at four stages and replicates in the three genotypes.

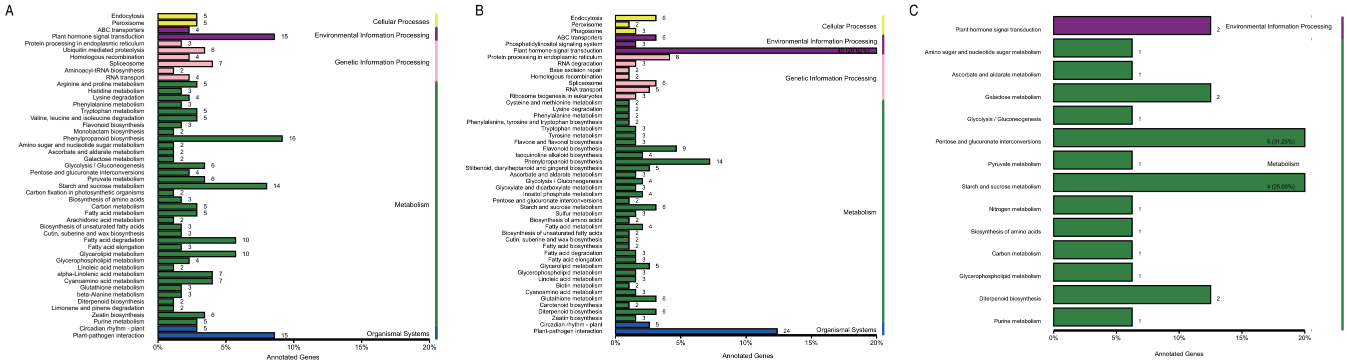

Figure S6. KEGG classification of A. cyan module, B. paleturquoise module and C. darkturquoise module.
